# Multiple free dorsal flaps for symmetrical peripheral gangrene of the extremities: A case report

**DOI:** 10.1016/j.jpra.2022.04.001

**Published:** 2022-04-28

**Authors:** Shoichi Ishikawa, Haruno Miyazaki, Shigeru Ichioka

**Affiliations:** Department of Plastic, Reconstructive and Aesthetic Surgery, Saitama Medical University, 38 Morohongo, Moroyama, Iruma-gun, Saitama 350-0495, Japan

**Keywords:** Symmetrical peripheral gangrene, Multiple free flaps, Scapular flap, Parascapular flap, Latissimus dorsi flap, Thoracodorsal artery perforator flap

## Abstract

We present a case of symmetrical peripheral gangrene of the extremities due to acute infectious purpura fulminans that was reconstructed with four free flaps harvested from the bilateral backs. We reconstructed the right and left, upper and lower limbs using the parascapular flap and latissimus dorsi muscle flap from one side and the scapular flap and thoracodorsal artery perforator flap from the other side, in multiple stages. All four flaps survived, preserving the right and left heels and function of the bilateral wrist joints. Although there have been several reports of single-stage elevation of the combined scapular and latissimus dorsi flaps, there has been no report of multi-stage elevations of these flaps. When multiple flaps are required in multi-stage, raising the flaps based on the thoracodorsal artery and scapular circumflex artery from the ipsilateral back is a useful method because it does not require additional donor sites.

## Introduction

There have been several reports of combined scapular and latissimus dorsi flaps.^1^^,^^2^ However, there has been no report of elevation of these flaps in multi-stage operations. We report a case of symmetrical necrosis of the extremities due to acute infectious purpura fulminans in which four amputated stumps were reconstructed by the bilateral thoracodorsal artery flaps and scapular circumflex artery flaps in multiple stages.

## Case report

**Fig. 1:**
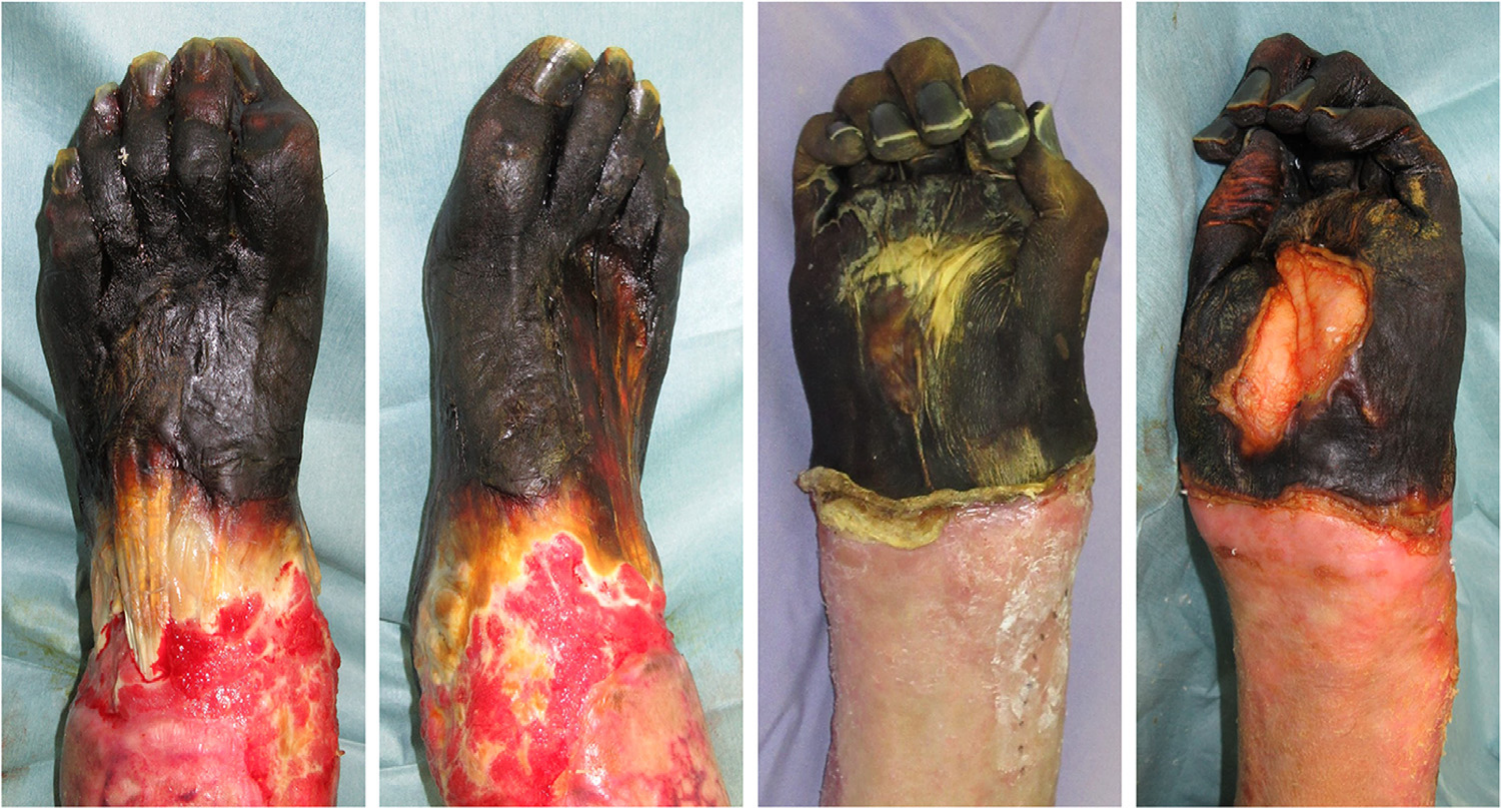
Preoperative appearance. Black necrosis of the peripheral areas of the right and left limbs.

The patient was a 52-year-old male with no particular history of illness. Three months prior to his admission to our hospital, he developed meningococcal sepsis and received medical treatment at another hospital. During the medical treatment, blood flow disturbance at the ends of the extremities occurred and eventually resulted in black necrosis (Fig. 1). After his general condition improved, he wanted to keep his legs and hands as long as possible, so the hospital where he received medical treatment referred him to our hospital.

First, due to the presence of infection, debridement of both feet was performed. After resection of the black necrotic tissue, it was determined that the calcaneus could be preserved and there was a high possibility of lower limb salvage. Therefore, reconstruction with free flap was planned. Angiography of the upper and lower extremities was performed, and it was confirmed that blood flow to the proximal side was good.

**Fig. 2:**
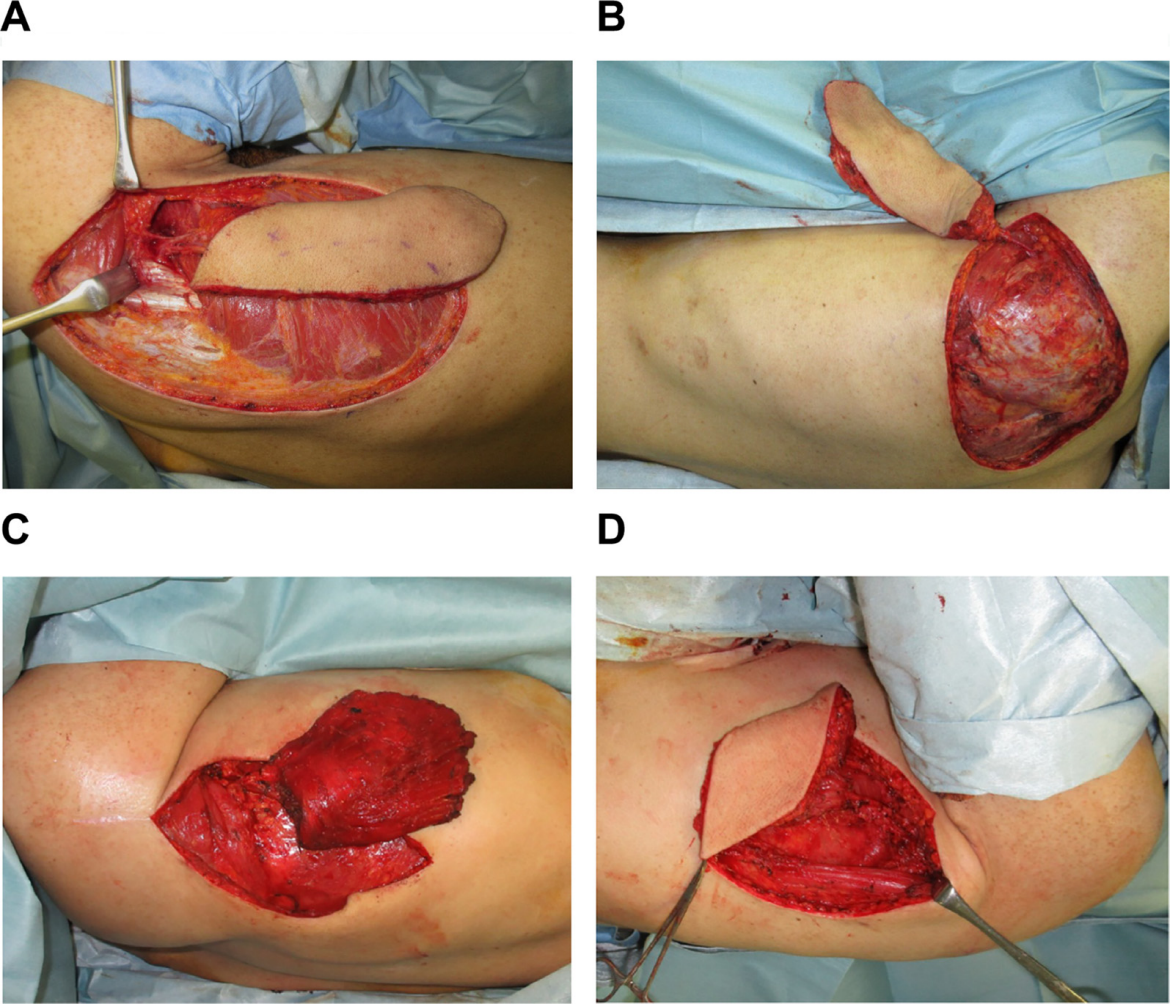
Intraoperative view of flap harvesting. A. Right parascapular flap B. Left scapular flap C. Right latissimus dorsi muscle flap D. Left thoracodorsal artery perforator flap.

Two weeks after the debridement surgery, reconstruction of the left foot was performed in the left lateral recumbent position. The left foot was amputated at the Chopart joint and reconstructed it with a 19 × 8.5-cm right parascapular flap (Fig. 2A). The recipient artery was the left posterior tibial artery. Two weeks later, reconstructive surgery of the right foot was performed in the right lateral recumbent position. The right foot was amputated at the Chopart joint and reconstructed with a 17 × 9-cm left scapular flap (Fig. 2B). The recipient artery was the right posterior tibial artery. Both flaps in the right and left legs survived without necrosis.

One month later, the left hand was operated on in the left lateral recumbent position. The left hand was amputated at the metacarpal bone and reconstructed with a 12 × 12- cm right latissimus dorsi muscle flap and skin graft (Fig. 2C). The recipient artery was the left radial artery. Three weeks later, the right hand was operated on in the right lateral recumbent position. The right hand was amputated at the metacarpal bone and reconstructed with a 10 × 7.5-cm left dorsal thoracic artery perforator flap (Fig. 2D). The recipient artery was the right radial artery. The flaps in both hands survived without necrosis, but the skin graft on the muscle flap implanted in the left hand took longer to take.

**Fig. 3:**
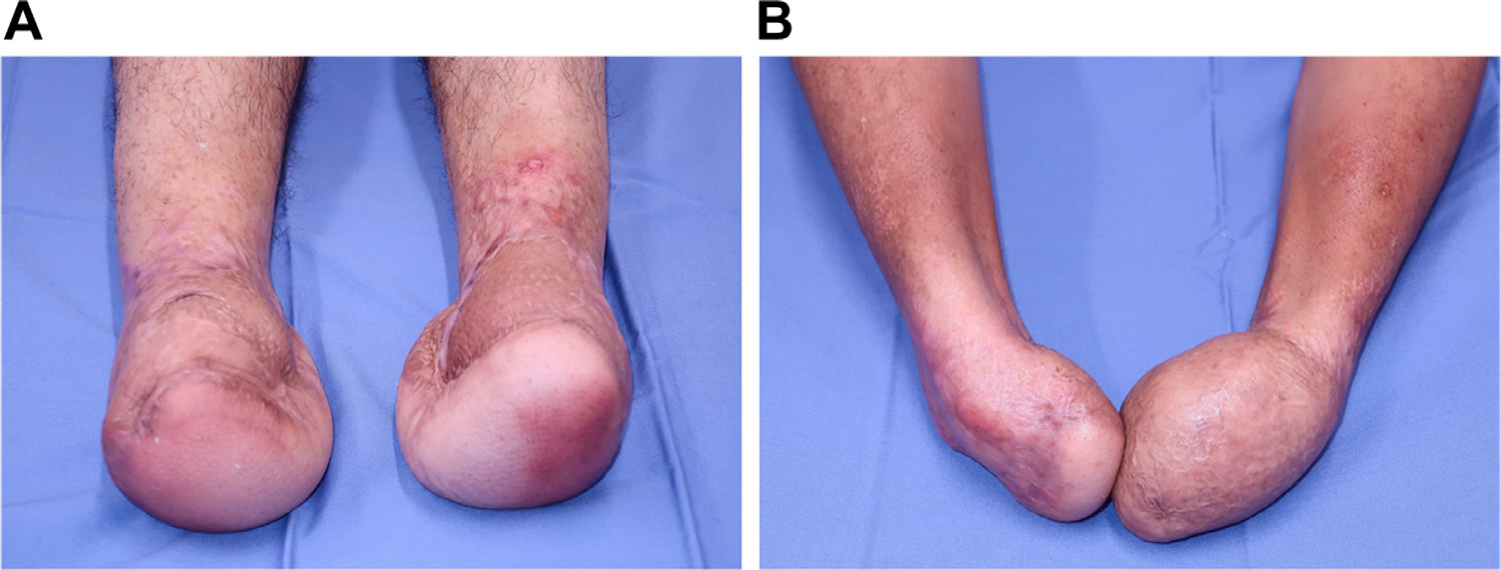
Appearance 1.5 years after surgery. A Bilateral heels are preserved. B Function of bilateral wrist joints are preserved.

One and a half years have passed since reconstructive surgery, and he is still able to walk with leg braces on his lower limbs **(**Fig. 3A). He also wears prosthetic hands for his upper limbs and is able to eat with a cutlery and operate a smart phone (Fig. 3B).

## Discussion

Symmetric peripheral gangrene presents as symmetrical gangrene of two or more limbs without occlusion of the great vessels or vasculitis. There are many causes of this disease, and the one caused by sepsis due to bacterial infection is called acute infectious purpura fulminans.^3^ Extensive necrosis may require major amputation, but there have been reports of patients who avoided major amputation by implanting a free flap at the amputation stump.^4^^,^^5^

In this case, the patient had the option of lower leg amputation for the lower limb and forearm amputation for the upper limb. However, the patient wanted to keep the limb as long as possible. Therefore, we resected only the necrotic tissue and reconstructed the extremities with four free flaps. As a result, we were able to preserve the calcaneus and wrist joint.

For foot reconstruction, we use scapular and parascapular flaps, which have thicker skin to withstand loading and the wearing of a leg braces. We choose a scapular flap as the first choice for foot reconstruction, and a parascapular flap if the defect is large. There are other reports of foot reconstruction using an anterolateral thigh flap^6^^,^^7^ and a deep inferior epigastric artery perforator flap^8^ as a thin cutaneous flap. However, harvesting the anterolateral thigh flap causes thigh pain and muscle weakness, and the inferior abdominal deep artery perforator flap has skin thinner than the back skin.

Reconstruction of the hand was performed with a muscle flap because it was thin and supple. With the advantage of not creating a new donor, the incision through which the parascapular flap was harvested was reopened and the latissimus dorsi muscle flap was elevated. However, it took a long time for the skin graft on the muscle flap to take. Therefore, we reconstructed the other hand with a thoracodorsal artery perforator flap, which does not require a skin graft.

When multiple flaps are needed in multi-stage, it is useful to elevate the flap based on the thoracodorsal artery and the scapular circumflex artery from the ipsilateral back. If there is a possibility of harvesting the multiple flaps from the back, it is advisable to harvest the first flap as peripherally as possible in the thoracodorsal artery or the circumflex scapular artery instead of harvesting it in the subscapular artery. As a result, the other flap can be preserved and used later, eliminating the need for a new donor site.

## Data statement

Not applicable.

## Funding statement

None.

## Ethics approval statement

Not necessary as this is a case report.

## Informed consent and patient details

Written consent for publication was obtained.

## Author contributions

Conceived and designed the analysis: Shoichi Ishikawa

Data collection: Shoichi Ishikawa, Haruno Miyazaki

Contributed data/analysis tools: Shoichi Ishikawa

Performed the analysis: Shoichi Ishikawa

Wrote the paper: Shoichi Ishikawa, Haruno Miyazaki, Shigeru Ichioka

## Declaration of Competing Interest

None.
